# How Does Paternal Odor Influence Perception of Fearful and Happy Faces in Infancy?

**DOI:** 10.1111/infa.70081

**Published:** 2026-03-12

**Authors:** Antonia Düfeld, Sarah Jessen

**Affiliations:** ^1^ Institute of Medical Psychology Center of Brain, Behavior, and Metabolism University of Lübeck Lübeck Germany

**Keywords:** EEG, emotion, face processing, father, infancy, social odor

## Abstract

Social odor plays an important role for various facets of early development, including communication and social processing. Previous research focusing on maternal odor has shown that smelling the mother can influence face processing in general as well as emotion processing more specifically. However, it is unclear to what extent these effects are specific to maternal odor or can also be found for other familiar social odors. To address this question, we investigated the impact of the father's odor on emotional face processing in 7‐month‐old infants (age at appointment 1: 209 ± 6 days [mean ± SD], range: 199–225 days; age at appointment 2: 217 ± 6 days, range: 206–231 days; gender: 15 girls and 15 boys). We recorded the infant's EEG response to female and male happy and fearful faces while infants were exposed to either their father's odor or the odor of a different infant's father. Analysis of the frontocentral Nc amplitude revealed an enhanced response to fearful compared to happy male faces only when infants smelled their own father but not when they smelled an unfamiliar father. In contrast, emotion processing at the occipital N290 was not affected by the presence of paternal odor, suggesting an impact of social odor on attention allocation rather than structural face processing. Interestingly, all effects were specific to male faces, pointing to a gender‐specific impact of social odor. Our findings therefore provide first evidence for an influence of the father's odor on face processing, specifically male faces, in infancy.

## 1 Introduction

Infants are born into a social environment and quickly learn to use social cues to gather information about their environment.

While numerous studies have investigated infants' processing of facial and vocal information, only in recent years, researchers have started to study olfaction as a source of social information in infancy. First studies in newborns focused primarily on the role maternal odor can play in the context of stress regulation (Kawakami et al. 1997; Nishitani et al. 2009) and facilitation of breastfeeding (Sullivan and Toubas 1998), showing that unique chemosensory information available in the prenatal environment is being remembered (Schaal et al. 1998).

In contrast, studies in older infants between 3 and 12 months of age have primarily addressed the impact of maternal odor on different facets of face processing. Typically, these studies rely on the worn‐t‐shirt paradigm, in which the mother is asked to wear a standardized t‐shirt for a certain amount of time, and this t‐shirt is then used as a source of odor during the actual measurements. This paradigm is one of the most widely used methods for presenting body odors to infants in a controlled and replicable way. In a first study, Durand et al. (2013) demonstrated that maternal odor collected in such a way increases 4‐month‐old's looking time to faces (compared to non‐faces) and in particular leads to an increased attentional focus and direct gaze on the eye region. Furthermore, concurrent presentation of maternal odor (compared to no social odor) appears to facilitate face categorization; on a neural level, 4‐month‐olds show evidence for a stronger categorization of faces (Leleu et al. 2020) and face‐like figures (Rekow et al. 2021) but not objects (Rekow et al. 2020) when simultaneously smelling their mother compared to a scenario where they do not smell any social odor. This intersensory facilitation effect changes across infancy, continuously decreasing between 4 and 12 months of age (Rekow et al. 2024).

Interestingly, maternal odor not only influences attention to face and face categorization per se, but also has an impact on how information contained in faces is processed. For instance, when exposed to their mother's odor (compared to either an unfamiliar or no social odor), 7‐month‐olds showed a reduced neural response to fearful faces (Jessen 2020), suggesting that maternal odor impacts the processing of emotional information. Furthermore, both, maternal and unfamiliar social odor, affects 4‐month‐old's looking behavior toward familiar versus unfamiliar faces (Durand et al. 2020; Düfeld et al. 2025).

However, while all studies so far have investigated the role of *maternal* odor, most infants are raised also by other caregivers than the mother, among them often the father. The question therefore arises whether we can observe similar effects as previously reported for maternal odor also for paternal odor.

On the one hand, infants have more exposure to their mother's than to their father's odor. This is true for all infants since odor processing already develops prenatally, and after birth, infants show a preference for odor they were exposed to in the womb (Schaal 1988; Schaal et al. 1998; Schaal et al. 2020; Tristão et al. 2021). Furthermore, for many infants this increased exposure to maternal odor continues after birth, as many infants are breastfed, an activity strongly influenced by olfactory processes (Porter and Winberg 1999; Varendi et al. 1997) and necessarily linked to the mother. But even apart from breastfeeding, in many cultures, the mother is often the primary caregiver, spending on average more time with the infant than the father (Baildam et al. 2000; Harrison and Magill‐Evans 1996; Tikotzky et al. 2011). An increased exposure to the odor of the primary caregiver should lead to a higher familiarity, resulting possibly in a stronger impact on other types of social processing. In addition, since breastfeeding is an inherently positive experience (with not only a nutritious but also an emotion regulatory function, see e.g., Schaal et al. 2020; Schäfer and Croy 2023), maternal odor is not only more familiar but also has very strong positive associations.

On the other hand, social odor is a learned odor and continuously updated (Damon et al. 2021; Schaal et al. 2020; Sullivan and Opendak 2020), which is important, since a person's odor changes due to natural variations, for instance, in food intake and health status, but also due to artificial factors such as a new deodorant used. Importantly, previous studies investigating the impact of maternal odor on sociocognitive aspects such as face processing have not focused on a specific component of the mother's body odor (such as mamillary odor) (Durand et al. 2020; Rekow et al. 2024). Rather, they considered the entire odor of the mother, encompassing different types of body odor and potentially also artificial sources of odor such as deodorant in combination, as one social odor. Hence, infants need to constantly adapt their representation of their mother's odor, and there is no reason why postnatal learning and updating should be limited to one single odor and not also occur for other familiar odors, such as the father's odor. Hence, as the father is always accompanied by paternal odor (consisting of his body odor but also deodorant etc. typically used), just as the mother is accompanied with her specific odor, we could expect a similar learning process. While maternal odor may be more familiar, other social odor could be familiar enough to have a similar effect on sociocognitive processing.

As the familiarity of the odor on infant face processing may be of particular importance for emotion processing (Jessen 2020; Düfeld et al. 2025), our main aim was to investigate whether paternal odor impacts the neural processing of fearful faces in 7‐month‐old infants, as has been previously reported for maternal odor (Jessen 2020). At 7 months, infants typically show an enhanced response to fearful compared to happy facial expressions (see e.g., Leppänen et al. 2007; Peltola et al. 2009; Vaish et al. 2008), impacting ERP components linked to structural face processing (N290, P400; De Haan et al. 2003, 2007; De Haan et al. 2002) as well as attention‐allocation (Conte et al. 2020; Reynolds and Richards 2005). However, this response is influenced by a number of factors, including concurrent information in other sensory modalities (Jessen 2020), maternal anxiety (Bowman et al. 2022), and later attachment style (Peltola et al. 2025). In particular, social odor may influence emotion processing via (at least) two distinct but potentially simultaneously occurring mechanisms (see Düfeld et al. 2025): (1) It may facilitate face processing per se by providing additional sensory input, as suggested, for instance, by Rekow et al. (2024), resulting in improved face categorization but potentially also more efficient processing of information contained in the face, such as emotional expressions. (2) It may modulate the arousal system, acting as a safety signal, and thereby reduce the infant's response to negative signals, as has been argued by Jessen (2020).

To investigate whether paternal odor influences face processing as has previously been suggested for maternal odor, we conducted an EEG study, focusing our analysis on the ERP components Nc, N290, and P400 (see e.g., Dickey et al. 2021, for a recent overview). EEG recording and ERP analysis are particularly suited for these questions, as (1) EEG recordings are generally well tolerated by infants and allow for the investigation of early, rapid, and subtle changes; (2) the above‐mentioned ERP components are well‐established in the infant ERP literature, allowing for a direct comparison to prior work (Conte et al. 2020; Guy et al. 2016); and (3) analyzing ERPs allows us to differentiate between general, attention‐related processes indicated by changes in Nc amplitude and face‐specific effects, indicated by N290 and P400 amplitude.

In particular, the Nc, which can be observed at fronto‐central electrodes around 400–800 ms after stimulus onset, has been linked to attention allocation (Conte et al. 2020) in response to salient or interesting stimuli (Kungl et al. 2017; Nelson and De Haan 1997), and its sensitivity to faces is modulated by the infant's attentional state (Conte et al. 2020). In contrast, the N290 and P400, typically peaking at 290 ms respectively 400 ms after stimulus onset at occipital and parietal electrodes, have been linked to structural face processing (Di Lorenzo et al. 2020; Guy et al. 2016). Additionally, these components reflect infant's encoding of faces and are sensitive to face salience or familiarity (Conte et al. 2020). Taken together, the N290 and P400 indicate the perceptual encoding of faces, whereas the Nc reflects higher‐order attentional and emotional evaluation. With respect to emotion processing, all three components have been discussed and shown differential responses (Aran et al. 2023; Xie et al. 2019; Xie and Nelson 2021), yet provide complementary information. However, under which conditions which component is affected by which type of emotional information is still under debate.

Based on Jessen (2020), we expected infants to show a reduction in Nc response to fearful faces when exposed to their own father's compared to an unfamiliar father's odor.

One important aspect to consider is the gender of the face expressing the emotions. Not only do infants in many societies spend more time with their mother than their father, as mentioned above; they typically also see more female than male faces in general (Liu et al. 2015; Quinn et al. 2008; Rennels and Davis 2008). This increased familiarity with female faces has been suggested to result in a processing bias in favor of female faces (Quinn et al. 2002; Ramsey et al. 2005; Ramsey‐Rennels and Langlois 2006; Rennels et al. 2016; Righi et al. 2014), which has prompted developmental researchers to rely on female faces as stimulus material in many studies (see e.g., Aran et al. 2023; Vanderwert et al. 2015; Xie et al. 2019). Hence, we know little about the processing of emotions from male faces in infancy, and whether it differs from that of female faces. When investigating the role of paternal odor, however, it is essential to not only use female faces, as this would result in an inherent mismatch between olfactory and visual information. The second aim of our study is therefore to investigate the processing of male compared to female emotional facial expressions, and to what extent the impact of paternal odor is modulated by face gender.

## 2 Methods

Sample size and main analyses were preregistered and can be found here: https://aspredicted.org/CLG_DBM.

### 2.1 Participants

64 7‐month‐old infants were invited to participate in the study. Data of 30 infants (*age at appointment 1:* 209 ± 6 days [mean ± SD], range: 199–225 days; *age at appointment 2:* 217 ± 6 days, range: 206–231 days; 15 girls) were included in the final sample for the main analysis. 31 infants were excluded because they did not contribute at least 10 artifact‐free trials per condition at each appointment (*n* = 26), did not show up for the second appointment (*n* = 3), because of technical problems (*n* = 2), and an Nc amplitude more than 2 SD from the mean across all conditions (*n* = 3). Our sample size was estimated a priori based on a power analysis and pre‐registered in advance. Our planned sample size of *N* = 30 infants with useable data at both appointments allowed us to detect an effect of small‐to‐medium effect size (*f* = 0.22) with a type‐1‐error‐rate of 0.05 and a power of 0.8 (G*Power 3.1.9.7). “Useable” was defined as having at least 10 artifact‐free trials per condition, and data collection was stopped once the full sample size had been reached. The data was checked continuously following the recruitment process and data acquisition. For the corroborating analysis using general linear mixed models, we included all infants who contributed at least 10 artifact‐free trials for at least one appointment (*n* = 41).

Infants were recruited via the maternity ward at the local hospital, were born full‐term (gestation weeks 37–42), had a birth weight of at least 2500 g, and no known visual or neurological deficits or other known significant health problems.

Our sample was collected in Lübeck, a medium‐sized city in northern Germany (population approx. 220.000 inhabitants), where most families lived, with a smaller proportion living in surrounding rural areas. German was the main language spoken at home in most families. For the 33 infants contributing at least 10 trials per condition, all families consisted of a father and mother as the two main caregivers and either no sibling (*N* = 18), one sibling (*N* = 10), or two siblings (*N* = 5). Regarding the parents' educational background, 18.2% of the mothers had attained lower secondary education, 27.3% had upper secondary education, 24.2% held a bachelor's or equivalent degree, and 30.3% held a master's or equivalent degree. 24.2% of the fathers had lower secondary education, 27.3% had upper secondary education, 21.2% held a bachelor's or equivalent degree, and 27.3% held a master's or equivalent degree. We did not collect any information about participants' race or ethnicity, as this is not commonly collected in the country in which the study was conducted. In the community, the average household income is approximately 23.265 € (in 2023) (Statistik—Wachstum und Wirtschaftskraft 2023), 30% of the population have a migration background (defined as either foreigners, Germans through granting citizenship, or German‐immigrants), and in 2024, the mean age of mothers at the birth of their first child was 31.7 years with a lifetime birth rate of 1.1 children per woman (Burger et al. 2025).

The study was conducted according to the Declaration of Helsinki and approved by the ethics committee at the local University. Written informed consent was obtained from the guardians of the infant prior to data collection. Parents received a reimbursement of 35 Euro in total for their participation as well as a small toy for the infant. Testing was carried out at the laboratories on the campus of the local university.

### 2.2 Visual Stimuli

Infants were presented with a total of 24 colored photographs from the FACES database (Ebner et al. 2010), showing 6 women (actress‐ID: 010, 034, 040, 054, 063, 069) and 6 men (actor‐ID: 008, 049, 062, 066, 114, 167) between 19 and 31 years. Each person was shown once with a happy and once with a fearful expression, and expressions had been recognized with an accuracy above 90% in a prior rating study (Ebner et al. 2010).

### 2.3 Odor Manipulation

**FIGURE 1:**
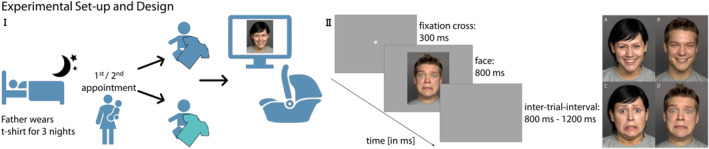
Experimental Set‐up and Design. (I) The fathers wore a t‐shirt for three nights in a row before coming to the experiment. At one appointment, infants were exposed to their father's odor, while at the other, they were exposed to a stranger's odor (order randomized). During the EEG recording, the t‐shirt was positioned over the infant's chest area while the infant was sitting in a car seat in front of a computer screen. (II) In the right part, a sample of one trial of the stimulus presentation as well as examples of all four stimulus categories are shown (A: female happy, B: male happy, C: female fearful, D: male fearful).

To manipulate the presence of paternal odor, we employed the “worn t‐shirt paradigm” (see Figure 1 for general procedure), in which paternal odor was captured by using worn cotton t‐shirts, as successfully used in previous studies (see e.g., Durand et al. 2013; Jessen 2020). Prior to the scheduled appointment, participating families were provided with a white, XL or XXL sized, 100% cotton t‐shirt in a zip‐lock bag. All t‐shirts were pre‐washed in the same way with a skin‐friendly and unscented detergent (Persil Sensitive Gel). The infant's fathers were asked to wear the t‐shirt during sleep for three consecutive nights, storing it in the zip‐lock bag throughout the day. Fathers had to indicate the dates the t‐shirt was worn during sleep to ensure that all fathers complied with wearing the t‐shirt as instructed. Additionally, the fathers were instructed to use their usual toiletries and refrain from using any new soap, perfume, etc. or change their eating and drinking habits during these days. After odor exposure, the t‐shirt was stored in a household freezer to preserve its odor (Lenochova et al. 2009), only removed again on the testing date, and brought along to the experiment by the parent. Parents were asked to freeze the t‐shirt for at least 1 day, which all families did. For practical reasons and to further conserve the odor, we kept all t‐shirts in the lab's freezer (−16°C) between appointments to swap them between father‐infant‐dyads for the stranger odor condition. Each t‐shirt was used twice, once for the paternal odor condition for the father's own child and once for the stranger odor condition for a different infant. The t‐shirt used in the father condition was not previously used in the stranger condition.

### 2.4 Design

The experiment followed a within group design (2 × 2 × 2 design) with the factors Odor (father, stranger), Emotion (happy, fear), and FaceGender (male, female). Infants were tested on two separate EEG appointments within 4 week's time. All infants were exposed to their father's odor on one appointment and a stranger's odor on the other appointment, with the order being counterbalanced across participants.

### 2.5 Procedure

Prior to the lab visit, participating families were sent a set of four questionnaires and asked to fill them in at home and bring them along for the first appointment: the German short version of the Infant Behavior Questionnaire (IBQ‐R) (Gartstein and Rothbart 2003; validated in Vonderlin et al. 2012), the Edinburgh Postnatal Depression Scale (EPDS) (Cox et al. 1987), and the Early Motor Questionnaire (EMQ) (Libertus and Landa 2013), and a lab internal questionnaire (LAB‐Q), obtaining information on parameters pertaining to the infant and their environment. Relevant to the current analysis, parents were asked (a) whether the infant had ever been, and if so, was still, breastfed, and (b) how many hours the mother respectively the father spend with the infant on an average day (i.e., within 24 h, combining day‐ and nighttime). The EPDS was collected to ensure mothers did not have an elevated depression score. One mother, in fact, had an elevated score of 15; however, exploratory excluding her from the analyses did not change the pattern of results; hence, we decided to retain the infant's data in the final sample.

All measurements in the lab were carried out by two female experimenters, who refrained from using perfume or perfumed products on the day of the testing to avoid odor contamination during the experiment. Upon arrival at the lab, families and infants were given time to familiarize themselves with the lab environment and the two experimenters. Parents were informed about the exact procedure of the experiment, had the opportunity to ask questions, and signed a consent form. For all except one appointment, the infant was accompanied by their mother, sometimes additionally by the father or other family members.

Testing took place in a light‐attenuated room, with consistent conditions (closed blinds and dimmed light) maintained across sessions to minimize external variability. The room was thoroughly aired between each measurement session.

Preparation for the EEG recording was done while the infant was sitting on their parent's lap. An elastic cap (BrainCap, Easycap GmbH) with 27 AgAgCl electrodes arranged according to the international 10–20 system was used for recording, and skin‐friendly, slightly warmed EEG gel was applied to reduce impedances ideally below 20 kΩ. The EEG signal was recorded at a sampling rate of 500 Hz using a BrainAmp MR Plus amplifier and BrainVision Recorder Software (Brain Products). During the measurement, data were referenced to the Cz electrode.

After EEG preparation, the infants were seated in an age‐appropriate car seat (Maxi Cosi Pebble), which was placed on the lab's floor in a semi‐reclining position. For the father's odor condition, a t‐shirt worn by the father was placed over the infant's chest area, whereas for the stranger's odor, a t‐shirt worn by the father of one of the other infants was used. The t‐shirt was folded vertically and placed horizontally, with the armpit area facing toward the infant's chin as well as nose, making sure that the axillary area was as close to the chin as possible to maximize odor exposure. During the recording, the t‐shirt was maintained in the proper position and loosely secured by the seat's safety belts. If possible, both, the experimenter placing the t‐shirt and the accompanying parents, were blind to the odor condition, except in six cases, where this was not possible for practical reasons (such as parents having forgotten to bring the t‐shirt along for the first appointment). All other parents were not debriefed until after the second experimental session.

A 24‐inch monitor (resolution 1680X1050, refresh rate: 60 Hz) was positioned at a distance of 60 cm from the car seat at a height of approximately 35 cm from the ground to the bottom edge of the screen. Two loudspeakers were positioned on either side of the monitor. Furthermore, a small camera was placed on top of the screen to monitor the infant's attention and to exclude any trials in which the infant was too inattentive or did not look at the screen.

The experiment was implemented using the software Presentation (version 22.1). Images were presented in isolation at the center of the screen on a gray background at a size of 24 × 30 cm. A trial started with a fixation cross, shown for 300 ms, followed by the face stimulus for 800 ms, and a jittered inter‐trial interval of 800–1200 ms (see Figure 1). At each appointment, the infants saw a maximum of 216 trials, arranged in nine blocks of 24 trials, consisting of the 24 different face stimuli (12 happy; 12 fearful). Within a block, the 24 stimuli were presented in pseudo‐randomized order, ensuring that none of the stimulus categories (female happy, female fearful, male happy, male fearful) was repeated more than once. The order of visual stimuli was randomized for each infant and for each of the two measurement appointments. To redirect the infant's attention to the screen, colorful, dynamic video clips accompanied by ringtones could be presented by the experimenter as attention‐getters whenever the infant looked away.

Out of the 33 infants contributing at least 10 trials per condition, 20 watched the whole set of 216 trials, while for 13 of them the experiment was stopped after a mean of 177 trials at appointment one (SD = 36 trials) due to fussiness or crying. During appointment two, 21 infants watched the whole set, while for 12 of them the experiment ended after a mean of 153 trials (SD = 44 trials).

During the measurement, the mother (in one case the father) remained in the same room but was seated approximately 1.5 m behind the infant, so as not to influence the testing or distract the infant from the visual stimuli. The parent was instructed not to interact or engage with the infant during the experiment, and one experimenter remained in the room at all times. If the father also came along to the appointment, he waited in an adjacent room during the experiment, except for the one case, where the father was in the room while measurement took place, as he was the only accompanying parent.

For the father's odor session, infants sat in front of the computer screen for a mean of 10.69 min (SD = 1.17 min) and watched the stimuli presentation, while they sat for a mean of 10.96 min (SD = 0.89 min) in the stranger condition. There was no statistically significant difference in exposure duration between the father and stranger session (*t*(31) = −1.18; *p* = 0.25, *d* = −0.21 [Father < Stranger]).

### 2.6 EEG Processing

Preprocessing and further analysis of the EEG data was done using Matlab 2022b (The MathWorks Inc. Natick, MA) and customized scripts as well as the FieldTrip toolbox (Oostenveld et al. 2011).

All trials were segmented into one‐second epochs around the time window of interest (200 ms pre‐to 800 ms post‐stimulus onset). The data were re‐referenced offline to the linked mastoids, correspondingly the mean of TP9 and TP10, and the Cz signal was reconstructed and treated like the rest of the electrodes. Subsequently, a bandpass filter was applied, ranging from 0.2 to 20 Hz. All electrode channels deviating more than two standard deviations from the mean in at least 50% of the segments were identified and interpolated using spherical spline interpolation; this was the case for at least one electrode in 26 out of 60 data sets.

After interpolation, all trials in which the standard deviation exceeded 80 μV at any electrode in a sliding window of 200 ms were excluded from further analysis. The remaining data were inspected visually to screen for any remaining artifacts. At this point, an average of 115 trials (SD = 36 trials) across all infants remained. Finally, any trials in which the infant did not attend to the screen based on the video recording were excluded from further analysis, retaining an average of 97 trials (SD = 33 trials) across all infants and conditions. The percentage of trial attrition at these two preprocessing stages is around ∼16%. Infants had to provide a minimum of at least 10 artifact‐free trials per condition (female‐happy, male‐happy, female‐fearful, male‐fearful) at each of the two appointments to be included in the final sample for the main analysis (see Table 1).

**TABLE 1:** Trial information.

	Female happy	Female fearful	Male happy	Male fearful
Total	24 ± 9	25 ± 9	24 ± 8	25 ± 9
Father odor	25 ± 9	26 ± 9	24 ± 8	24 ± 10
Stranger odor	23 ± 8	24 ± 9	24 ± 8	25 ± 8

*Note:* Overview of the mean trial number in total across conditions and based on odor exposure (father and stranger) for infants included in the final sample. Shown are mean ± standard deviation.

### 2.7 ERP Analysis

For the main statistical analysis, initially data from a total of 33 7‐month‐old infants were included.

Following preprocessing, the data were statistically analyzed in Matlab (version 2022b) and Jamovi (version 2.3.28). As preregistered, the Nc response was analyzed at frontocentral electrodes (F3, Fz, F4, C3, Cz, C4) in a time window of 400–800 ms after stimulus onset. The N290 was analyzed in a time window of 100–300 ms and the P400 in a time window of 300–500 ms after stimulus onset at occipital electrodes (O1, O2). In addition to the preregistered time windows, we also analyzed all components in time windows based on visual inspection after peak analysis across all the conditions and participants (referred to as preregistered vs. exploratory analysis in the Results).

For Nc, N290, and P400, mean responses were computed over the respective time windows and electrodes. Three participants had a mean Nc amplitude of more than 2 standard deviations from the mean across all conditions, and were excluded from further analysis. Data from the remaining 30 participants were entered into a repeated measures ANOVA with the within‐subject factors Emotion (happy, fearful), FaceGender (female, male), and Odor (father, stranger). As post‐hoc tests, Student's *t*‐tests are computed. Effect sizes are reported as partial eta squared (*η* ^*2*^ *p*) and Cohen's *d*.

In addition, and to corroborate the findings from the repeated measures ANOVA in a larger sample, we computed a general linear mixed model, which allowed us to include the data from infants who only contributed a sufficient number of trials at one appointment. Here, we did not exclude entire participants as outliers but only those data points which were more than 2 standard deviations from the mean (i.e., we excluded single conditions for individual infants). Using the GAMLj package (version 2.4.0) in jamovi, we computed the following model (plus the interactions between the three factors) for all three ERP components:

ERPamplitude∼1+Odor+FaceGender+Emotion+(1|SubjectID),

where SubjectID was included to account for interindividual variance.

### 2.8 Transparency and Openness

We report how we determined our sample size, all data exclusions, all manipulations, and all measures in the study, and we follow JARS (Appelbaum et al. 2018). The data that support the findings of this study will be made available on OSF after publication. All data were analyzed using Matlab, version 2022b, and Jamovi version 2.3.28. This study's design and its analysis were pre‐registered (https://aspredicted.org/CLG_DBM).

## 3 Results

### 3.1 Sample

Infants in the main sample spent on average 21.0 ± 3.04 (mean ± standard deviation) hours/day (out of 24 h), with their mother and 9.29 ± 4.26 h/day with their father, and all infants spent more time with their mother than with their father. At the time of the first appointment, 22 (out of 30) infants were still breastfed.

### 3.2 ERP Analysis

We computed a repeated measures ANOVA with the factors Odor (father, stranger), Emotion (happy, fearful) and FaceGender (female, male) for the Nc, the N290 and the P400.

**FIGURE 2:**
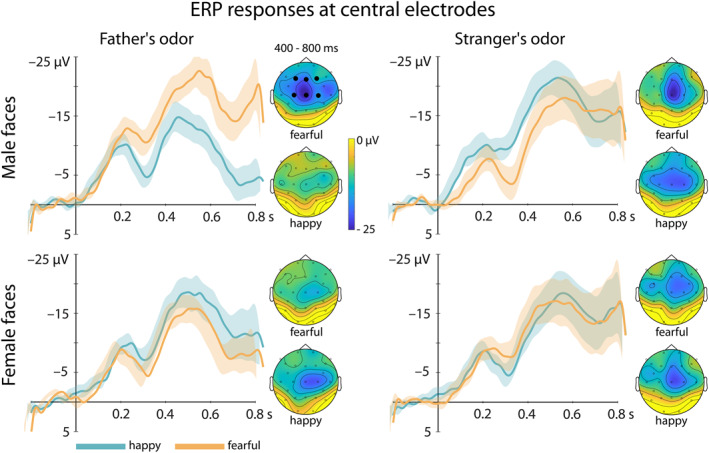
ERP responses at the central electrodes. Shown are the ERPs for the Nc response at frontocentral electrodes (F3, Fz, F4, C3, Cz, C4; marked by black dots) to male and female faces in the two emotion conditions, happy (blue) and fearful (orange), in both the father's odor condition (left panel) as well as the stranger's odor condition (right panel). The ERP responses are plotted with negative polarity being up. The shaded ribbon of the waveform represents the within‐subject standard error (SEM). While no difference in response was observed for female faces and the stranger's odor condition, infants show an enhanced Nc response to male fearful compared to male happy faces in the father's odor condition. Next to the ERP curves, topoplots of the EEG signal across all electrodes averaged in the time window of interest (400–800 ms) post stimulus onset are presented.

For the Nc, we observed an interaction between all three factors (*F*(1,29) = 6.48, *p* = 0.016, *η*_*p*_^2^ = 0.18; see Figure 2), revealing an interaction between Emotion and Odor for male (*F*(1,29) = 7.99, *p* = 0.008, *η*_*p*_^2^ = 0.22) but not for female faces (*F*(1,29) = 0.61, *p* = 0.44, *η*_*p*_^2^ = 0.02). When smelling their father, infants showed an enhanced Nc response to fearful male compared to happy male faces (*t*(29) = 2.45, *p* = 0.021, *d* = 0.45, fearful = −18.48 ± 17.5 μV [mean ± SD], happy = −8.55 ± 12.9 μV). When smelling a stranger, we did not find a difference in response between the two emotions (*t*(29) = −0.99, *p* = 0.33, *d* = −0.18).

In addition to the pre‐registered analysis of the Nc, we performed an exploratory analysis in time windows of 300–700 ms as well as 420–600 ms to more specifically cover the Nc observed in the present data across conditions. We observed the same effects as in the preregistered time window of 400–800 ms.

**FIGURE 3:**
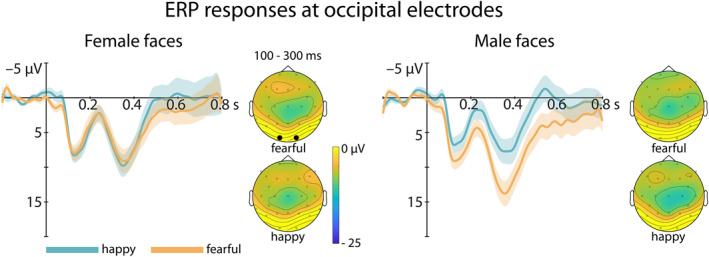
ERP responses at occipital electrodes. Shown are the ERPs for the N290 and P400 responses at occipital electrodes (O1, O2; marked by black dots) to male and female faces in the two emotion conditions, happy (blue) and fearful (orange), independent of the odor condition. The responses are plotted with negative polarity up. The shaded ribbon of the waveform represents the within‐subject standard error (SEM). Infants showed a more positive amplitude in response to fearful compared to happy male faces. Next to the ERP curves, topoplots of the EEG signal across all electrodes averaged in the time window of interest (preregistered 100–300 ms) post stimulus onset are presented. See Supplementary Material for split by Odor (see Supporting Information S1: Figure S1).

For the N290, we observed an interaction between FaceGender and Emotion (*F*(1,29) = 5.149, *p* = 0.031, *η*_*p*_^2^ = 0.15; see Figure 3), but this interaction was not further influenced by Odor (*p* > 0.23). Only for male faces, infants showed a larger N290 amplitude for happy compared to fearful faces (*t*(29) = −2.13, *p* = 0.042, *d* = −0.39, fearful = 7.00 ± 7.17 μV, happy = 3.78 ± 6.24 μV).

Exploratorily, we also included P7 and P8 in the analysis of the N290 and P400 (see Guy et al. 2016; Conte et al. 2020, who also included more parietal electrodes in their analysis of these components). Furthermore, we redefined our time window for the N290 based on visual inspection of the ERP response across all conditions and analyzed the amplitude in a time window of 190–290 ms after stimulus onset. While the repeated measures ANOVA suggested an interaction Odor* Emotion*Gender (*F*(1,29) = 4.64, *p* = 0.040, *η*_*p*_^2^ = 0.14), post‐hoc tests turned out non‐significant. Including hemisphere (O1, P7 vs. O2, P8) and ROI (O1, O2 vs. P7, P8) in the analysis did not affect the results.

For the P400, we observed a marginally significant interaction between FaceGender and Emotion (*F*(1,29) = 4.16, *p* = 0.051, *η*_*p*_^2^ = 0.13; see Figure 3) between 300 and 500 ms as defined a priori. For male faces, infants showed a more positive P400 for fearful compared to happy faces (*t*(29) = −2.12, *p* = 0.042, *d* = −0.39, fearful = 10.3 ± 10.9 μV, happy = 5.4 ± 10.6 μV). In the exploratory time window of 280–430 ms based on visual inspection, we observed the same pattern of results (FaceGender*Emotion: *F*(1,29) = 4.364, *p* = 0.046, *η*_*p*_^2^ = 0.013; male faces: *t*(29) = −2.05, *p* = 0.050, *d* = −0.37, fearful = 8.55 ± 10 μV, happy = 4.30 ± 9.7 μV; female faces: *t*(29) = 0.42, *p* = 0.677, *d* = 0.08, fearful = 4.93 ± 7.2 μV, happy = 5.59 ± 9.2 μV).

Including various potential confounds in the analysis did not affect the results. In particular, we did not find an influence of: (1) breastfeeding experience (Breastfeeding [yes/no], coding whether the infant was still breastfed at the time of the first appointment); (2) time spent with the father (*z*‐scored); (3) IBQ‐R or EMQ scores; (4) infant sex.

To corroborate these analyses, we computed a linear mixed model for the larger sample of infants (i.e., *n* = 41, including all infants who contributed 10 trials per condition for at least one appointment). Our model yielded the same results for the interaction FaceGender*Odor*Emotion on the Nc (*b* = −12.20, SE = 5.88, *t*(261.5) = −2.08, *p* = 0.039), the interaction FaceGender*Emotion on the N290 (*b* = 4.05, SE = 2.05, *t*(254.5) = 1.98, *p* = 0.048) and the interaction FaceGender*Emotion on the P400 (*b* = 4.94, SE = 2.75, *t*(263.0) = 1.79, *p* = 0.074), confirming results from the repeated measures ANOVA in the increased sample.

## 4 Discussion

We investigated the impact of the father's odor on the processing of fearful and happy facial expressions in 7‐month‐old infants. Infants who smelled their father showed an enhanced Nc response to male fearful compared to male happy faces, while no difference in Nc response was observed for either female faces or infants smelling a different infant's father. Furthermore, on the N290 and P400 response, we found no influence of paternal odor but infants showed a more positive amplitude in response to fearful compared to happy male but not female faces.

### 4.1 Paternal Odor Enhances Nc Response to Fearful Male Faces

As predicted, the presence of paternal odor had an impact on emotion processing in 7‐month‐old infants by influencing the Nc response to fearful faces. Hence, paternal odor does influence sociocognitive processing in infancy, as had previously only been shown for the mother's odor (Durand et al. 2020; Jessen 2020). This effect was specific to the Nc component and not observed at the occipital components N290 or P400. As the Nc has been linked to attention allocation (Ackles and Cook 2007; Conte et al. 2020), this suggests that paternal odor affected attention related processes; smelling the father prompted infants to pay more attention to specifically male faces. While purely speculative at this point, one explanation could be that infants paid more attention trying to match the familiar paternal odor to one of the male faces, that is, to detect their father among the several male identities shown. In contrast, in the stranger odor condition, both, odor and faces were unfamiliar. Along these lines, it has been suggested that the Nc reflects the integration of multisensory information, specifically as it relates to attention, salience, and intersensory processing (Reynolds et al. 2014), which is necessary to link olfactory and visual information, as was the case in the present study.

Unlike for the Nc, we did not observe an impact of paternal odor on the N290 or P400, which are both rather linked to perceptual or structural processing of faces (Conte et al. 2020; De Haan et al. 2003, 2007; Guy et al. 2016). Hence, infants do seem to process faces per se irrespective of concurrent odor, and the impact of odor rather appears to affect later stages in processing. This interpretation is consistent with the role of the N290/P400 specifically in bottom‐up processing (Di Lorenzo et al. 2020; Guy et al. 2016; Hadders‐Algra 2022; Nava et al. 2023), focusing on the perceptual features of the face itself, without being substantially influenced by contextual information such as odor.

Interestingly, this effect was specific for male faces, and did not modulate processing of female facial expressions. Several explanations for this pattern are possible. While it has previously been suggested that infants learn to associate faces with odor (Leleu et al. 2020; Rekow et al. 2020), thereby facilitating face categorization, it may be the case that this association is gender‐specific. If infants have learned to associate male faces with male odor and female faces with female odor, paternal odor might specifically influence the processing of male faces. Since social odor differs markedly between men and women (Mutic et al. 2016; Penn et al. 2007; Russell 1976; Troccaz et al. 2008), it seems plausible that infants may also be sensitive to this difference.

Another explanation could be general processing differences between male and female faces. While we did not find an effect of paternal odor on occipital face processing, also here, we only observed differential emotion processing for male faces. This suggests a more general difference in emotion processing between male and female faces, irrespective of odor. Since most prior studies investigating emotional face processing in infants used only female faces (Aran et al. 2023; Vanderwert et al. 2015; Xie et al. 2019) or did not systematically compare the processing of male and female faces (Hoehl and Striano 2008, 2010), we know little about how infants process emotions from male faces. However, since infants show a processing bias for neutral female faces (e.g., Marquis and Sugden 2019; Righi et al. 2014), it is likely that infants also process emotional information differently.

A likely interpretation of the present pattern of results is therefore, that infants overall showed a structural differentiation between fearful and happy male faces, which was further modulated at an attentional level by the presence of paternal odor.

### 4.2 Absence of Differential Emotion Processing When Smelling an Unfamiliar Father

An interesting difference to previous work using maternal odor is the fact that we found an enhanced response to fearful faces in the presence of the father's but not a stranger's odor, while the reverse pattern has been reported for maternal odor (i.e., an enhanced response to fearful faces only in the presence of a stranger's but not the mother's odor, Jessen 2020).

One explanation for this different pattern could be differences in odor familiarity. Infants in our sample spent on average twice as much time with their mother compared to their father (20.9 vs. 9.21 h/day), making the mother the primary caregiver. Hence, while the father's odor certainly is familiar (as shown by the reported odor effect), it is likely less familiar than the mother's odor and hence might have a different impact on other social processes. This experience‐driven account would be in line with research on face processing, which suggests that the amount of time infants spend with their father impacts their processing of male versus female faces (e.g., Gredebäck et al. 2012; Liu et al. 2015). It might be the case that the father's odor draws attention but does not have the same buffering effect as maternal odor due to lower exposure. In this respect, the observed difference would be explained by different responses to primary versus secondary caregiver rather than maternal versus paternal odor. We did not find direct evidence for such an exposure‐based explanation in our data—including time spent with the father in the model did not have an impact on the results. However, since in all cases, the mother spent more time with the infant than the father, the variance in the sample might be too small to detect an effect of exposure. Hence, future studies including infants with larger variation in parenting exposure and a larger overall sample could shed further light on an experience‐based account underlying an odor impact.

Another potential influence could have been the mother, who was present during the measurement. As in previous studies on maternal odor (e.g., Durand et al. 2020; Jessen 2020; Leleu et al. 2020), the mother was instructed to remain behind the infant at a distance to avoid any influence of her odor, but remained in the testing room at all times. However, in contrast to previous studies, this implied that the odor donor (i.e., the father) was absent, while in studies on maternal odor, the odor donor was in the room. This may have inadvertently caused a mismatch between the presence of one caregiver (the mother) while infants smelled the absent caregiver (the father), which in turn could impact concomitant face processing.

### 4.3 Absence of Differential Emotion Processing for Female Faces

While infants showed a differential response to fearful compared to happy male faces at both, occipital and central electrodes, we found no evidence for emotion discrimination from female faces. This lack of a discrimination effect contrasts with many prior studies who reported enhanced response to fearful female faces as central and/or occipital electrodes (e.g., Aran et al. 2023; Peltola et al. 2009; Xie et al. 2019) in comparable age groups. (Though note that these studies used visual stimulation without any odor exposure, while in the present study, faces were always presented in the presence of paternal/stranger odor.).

It may have been the case that—in addition to influencing the processing of male faces—paternal odor hampered the processing of female faces. If male odor is indeed associated with male faces, both, father's as well as stranger's odor, create a mismatch with female faces, which may have an impact on face processing, as has been reported for audiovisual emotion processing in infancy (Grossmann et al. 2006; Vogel et al. 2012).

Another interpretation might be that the use of male and female faces as stimulus material caused infants to pay more attention to male faces as the less familiar stimulus, which may have resulted in a reduced processing of information from female faces. However, such an account would contradict the assumed attentional bias for female face (Quinn et al. 2002; Ramsey et al. 2005; Rennels et al. 2016; Righi et al. 2014). Furthermore, prior studies using both, male and female faces, did report differential processing at central and/or occipital electrodes (Hoehl and Striano 2008, 2010), providing evidence against such an explanation (though the factor gender was not systematically investigated and the effect may have been driven by male or female faces).

### 4.4 Implications and Future Studies

Our results highlight the need to include social odor beyond the mother's odor in studying the impact of social odor in early development. We show that paternal odor (a) does have an impact on sociocognitive processing and (b) has an impact that is qualitatively different from the mother's odor. If we want to understand the overall influence of social odor in an infant's everyday life, this needs to be reflected in future research approaches.

To do so, future studies are needed to shed further light on the mechanisms behind the observed pattern. A first important step would be to directly compare the impact of maternal to that of paternal odor in the same sample of infants and using the same set of visual stimuli. Furthermore, a more systematic investigation of the infant's familiarity with male versus female faces in general and the time spent with mother versus father in particular is necessary to address the role of familiarity for the influence of paternal odor on face processing.

### 4.5 Conclusion

In recent years, several studies have explored the impact of maternal odor on sociocognitive processing in infancy. Here, for the first time, we provide evidence that this impact is not limited to maternal odor, but that paternal odor influences infant's processing of emotional faces as well. Interestingly, the observed effects were specific to male faces, and suggested a stronger emotion discrimination in the presence of paternal odor. Our results therefore provide an important first step for our understanding of the role different types of social odor play in early human development.

## Author Contributions

**Antonia Dufeld:** data curation, formal analysis, investigation, writing – original draft, writing – review and editing, visualization. **Sarah Jessen:** conceptualization, methodology, validation, resources, writing, supervision, funding acquisition.

## Funding

This work was supported by funding of the German Research Foundation (DFG, Grants JE 781/3‐1 and JE 781/4‐1).

## Ethics Statement

The study was conducted according to the Declaration of Helsinki and approved by the ethics committee at the local University. All procedures were performed in compliance with relevant laws and institutional guidelines. Written informed consent was obtained from the guardians of the infant prior to data collection. Privacy rights have been observed.

## Conflicts of Interest

The authors declare no conflicts of interest.

## Supporting information


**Figure S1:** ERP responses at the occipital electrodes.

## Data Availability

The data that support the findings of this study will be made available on OSF after publication. A link to the repository used will be made available.
