# Supplementary material for: How Does Paternal Odor Influence Perception of Fearful and Happy Faces in Infancy?
Source: Infancy. 2026 Mar 12;31(2):e70081. doi: 10.1111/infa.70081 (PMC12982682; doi:10.1111/infa.70081)
Supplement: Supplementary file 1 — Figure S1: ERP responses at the occipital electrodes. [file INFA-31-0-s001.docx]

**Supplementary Material**


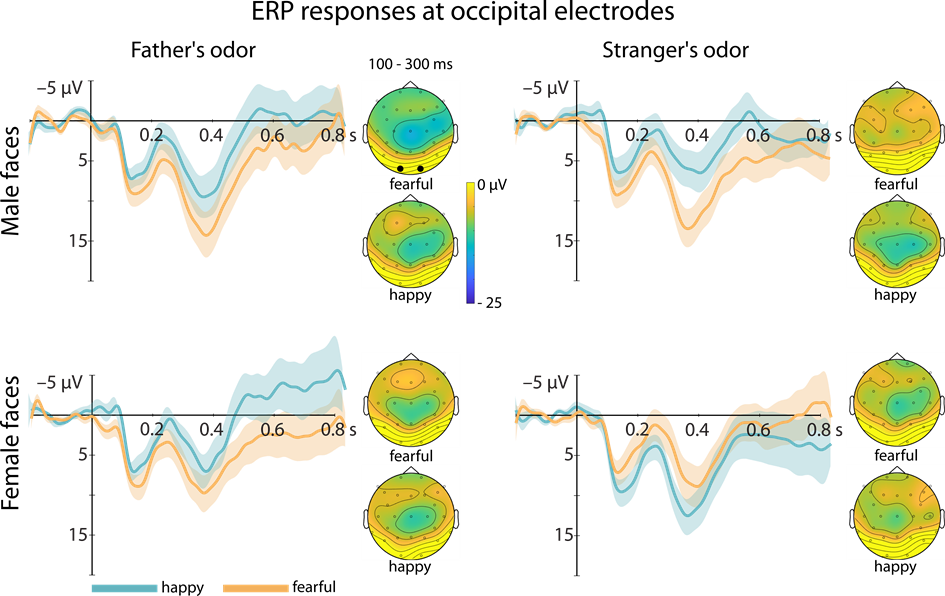


***Figure S1. ERP responses at the occipital electrodes.*** *Shown are the ERPs for the N290 and P400 responses at occipital electrodes (O1, O2; marked by black dots) to male and female faces in the two emotion conditions, happy (blue) and fearful (orange), in both the father’s odor condition (left panel) as well as the stranger’s odor condition (right panel). The responses are plotted with negative polarity up. The shaded ribbon of the waveform represents the within-subject standard error (SEM). For both components, there seems to be no odor effect, irrespective of familiarity. However, infants showed a more positive amplitude in response to fearful compared to happy male faces. Next to the ERP curves, topoplots of the EEG signal across all electrodes averaged in the time window of interest (100-300 ms) post stimulus onset are presented.*
